# Serum metabolomics of end-stage renal disease patients with depression: potential biomarkers for diagnosis

**DOI:** 10.1080/0886022X.2021.1994995

**Published:** 2021-11-01

**Authors:** Dezhi Yuan, Tian Kuan, Hu Ling, Hongkai Wang, Liping Feng, Qiuye Zhao, Jinfang Li, Jianhua Ran

**Affiliations:** a Department of Neurology, The Second Affiliated Hospital of Chongqing Medical University, Chongqing, China; b Department of Anatomy, and Laboratory of Neuroscience and Tissue Engineering, Basic Medical College, Chongqing Medical University, Chongqing, China; c Department of Nephrology, The Second Affiliated Hospital of Chongqing Medical University, Chongqing, China

**Keywords:** End-stage renal disease, depression, metabolomics, UPLC-QTOF/MS, biomarkers

## Abstract

**Background:**

End-stage renal disease (ESRD) is the final stage during the development of renal failure. Depression is the most common psychiatric disorder in patients with ESRD, which in turn aggravates the progression of renal failure, however, its underlying mechanism remains unclear. This study aimed to reveal the pathogenesis and to discover novel peripheral biomarkers for ESRD patients with depression through metabolomic analysis.

**Methods:**

Ultra-high-performance liquid chromatography coupled with mass spectrometry (UPLC-MS) was used to explore changes of serum metabolites among healthy controls, ESRD patients with or without depression. The differential metabolites between groups were subjected to clustering analysis, pathway analysis, receiver operating characteristic (ROC) curve analysis.

**Results:**

A total of 57 significant serum differential metabolites were identified between ESRD patients with or without depression, which were involved in 19 metabolic pathways, such as energy metabolism, glycerolipid metabolism, and glutamate-centered metabolism. Moreover, the area under the ROC curve of gentisic acid, uric acid, 5-hydroxytryptamine, 2-phosphoglyceric acid, leucyl-phenylalanine, propenyl carnitine, naloxone, pregnenolone, 6-thioxanthene 5'-monophosphate, hydroxyl ansoprazole, zileuton O-glucuronide, cabergoline, PA(34:2), PG(36:1), probucol and their combination was greater than 0.90.

**Conclusions:**

Inflammation, oxidative stress and energy metabolism abnormalities, glycerolipid metabolism, and glutamate-centered metabolism are associated with the pathogenesis of ESRD with depression, which may be promising targets for therapy. Furthermore, the identified differential metabolites may serve as biomarkers for the diagnosis of ESRD patients with depression.

## 1. Background

End-stage renal disease (ESRD) is the final stage of acute and chronic renal failure with accumulated metabolites and toxic substances, disordered electrolytes and acid-base balance, as well as some endocrine dysfunction, resulting in a series of autotoxic symptoms [1–4]. Depression is the most common psychological disorder, with a prevalence rate as high as 20–25% in patients with ESRD [5,6]. Depression has been identified as a complicating comorbid diagnosis in ESRD, shown as low mood, slow thinking, cognitive impairment, physical symptoms and even suicide [7–11]. There is overwhelming evidence that chronic illness with depression is associated with increased symptom burden and functional impairment, poor quality of life, nonadherence to treatment, and worse clinical outcomes [12–14]. Nevertheless, the pathogenesis of ESRD patients with depression remains unclear.

The kidney is a metabolically active organ involved in the handling of biochemical classes of metabolites [2,15–17]. One of the hallmarks of progression to ESRD is the plasma accumulation of certain metabolites and uremic solutes [18]. Therefore, the metabolomic profiling of patients with ESRD may be a promising method to identify new biomarkers for the prognosis of ESRD patients [19]. Ultra-performance liquid chromatography coupled with mass spectrometry (UPLC-MS) has high selectivity, high sensitivity, and good time-retention reproducibility, thereby suitable for metabolome analysis, especially for non-targeted metabolomics study [20–24]. Previous studies have demonstrated the dysregulation of various metabolites in CKD [18,25,26], while other studies have revealed that the levels of fatty acid metabolism, particularly the polyunsaturated fatty acids (PUFAs) metabolism, and purine metabolism, are significantly different between depressed and nondepressed patients [27]. However, there is no metabolomic study focusing on ESRD patients with depression to our best knowledge.

In this study, we aim to perform metabolomics analysis to discover new biomarkers for depression in ESRD and its possible underlying mechanisms.

## 2. Methods

### 2.1. Study populations

All participants had signed written informed consent papers before the study. The procedures were approved by the Ethics Committee of Chongqing Medical University. From January 2016 to July 2017, 17 ESRD patients without depression, and 17 ESRD patients with depression in the Department of Nephrology in the Second Affiliated Hospital of Chongqing Medical University were enrolled. The determination of depression was according to Hamilton Depression Rating Scale for Depression (HAMD) [28]: depression group with total score >17 points, and nondepression group with a total score ≤17 points. Hamilton Anxiety Scale (HAMA) was used to assess anxiety symptoms in subjects. Additionally, 12 healthy participants with matched age, gender, and body mass index (BMI) recruited from the physical examination center were included, and structured interview to exclude the psychiatric diagnosis.

Inclusion criteria were as follows: (1) Confirmed as chronic renal failure uremia; (2) Serum creatinine >707 mmol/L and endogenous creatinine clearance <15 mL/(min·1.73m^2^); (3) The diagnosis and treatment of the disease were informed; (4) With normal liver function and blood glucose fluctuation range of 5.4–11.2 mM; (5) Brain computed tomography and magnetic resonance imaging showed no new lesions; (6) No previous history of drug abuse, mental illness were all excluded.

### 2.2. Sample and clinical data collection

The blood is collected before the patient starts the dialysis course and all participants underwent venous blood collection 10 h after fasting. Collected blood was stored at 4 °C for 30–60 min followed by centrifugation at 3000 *g* for 10 min, and the supernatant was stored. The samples were stored at −80 °C with drikold as cold chain during transport. All participants underwent routine including height, weight, blood pressure, and BMI as well as blood tests. Plasma biochemical indicators for participants are shown in Table 1.

**Table 1.** Demographic and clinical characteristics of the three groups.

Indicators	Healthy control (*n* = 12)	ESRD patients without depression (*n* = 17)	ESRD patients with depression (*n* = 17)
Age (year)	57.00 ± 11.51	50.18 ± 10.65	50.82 ± 13.45
Hypertension history (month)	0.00 ± 0.00	73.82 ± 61.47**	66.76 ± 85.80*
Diabetes history (month)	0.00 ± 0.00	52.24 ± 88.69	43.06 ± 98.23
History of nephropathy (month)	0.00 ± 0.00	42.00 ± 38.29**	30.71 ± 43.41*
Heart disease history (month)	0.00 ± 0.00	0.29 ± 1.21	7.76 ± 16.40
SBP (mmHg)	126.33 ± 4.50	163.53 ± 23.15**	171.12 ± 33.06**
DBP (mmHg)	80.33 ± 2.67	94.24 ± 9.75**	101.59 ± 16.19**
Dialysis frequency (times/week)	0.00 ± 0.00	2.53 ± 1.01**	2.12 ± 1.11**
WBC (×10^9^/L)	5.79 ± 1.55	7.22 ± 2.53	6.62 ± 3.48
Neutrophils (%)	58.56 ± 8.17	69.23 ± 9.82*	78.24 ± 11.20^**△^
RBC (× 10^12^/L)	4.67 ± 0.35	3.73 ± 0.63**	3.17 ± 1.13**
Hb (g/L)	142.50 ± 13.36	109.71 ± 17.09**	92.29 ± 25.84**
TC (mmol/L)	4.72 ± 0.92	3.25 ± 1.57*	4.30 ± 1.68
TG (mmol/L)	1.25 ± 0.68	3.13 ± 1.50**	2.02 ± 1.46
Albumin (g/L)	44.88 ± 3.66	38.39 ± 2.92**	124.34 ± 280.64
Cr (μmol/L)	59.10 ± 12.43	985.87 ± 342.62**	915.14 ± 213.44**
BUN (mmol/L)	5.38 ± 1.43	21.37 ± 5.85**	28.72 ± 7.23^**△△^
K^+^ (mmol/L)	4.04 ± 0.51	4.78 ± 0.56**	4.67 ± 0.81*
Phosphorus (mmol/L)	1.09 ± 0.12	1.82 ± 0.47**	1.97 ± 0.82**
Ca^2+^ (mmol/L)	2.32 ± 0.15	2.15 ± 0.32	2.07 ± 0.33*
Anxiety scores	4.33 ± 5.66	7.41 ± 3.40	21.29 ± 4.91^**△△^
Depression scores	3.75 ± 3.11	9.00 ± 3.06**	22.88 ± 4.20^**△△^

SBP: systolic blood pressure; DBP: diastolic blood pressure; WBC: white blood cells; N: neutrophils; RBC: red blood cells; Hb: hemoglobin; TC: cholesterol; TG: triglycerides; Cr: serum creatinine; BUN: blood urea nitrogen. *p < 0.05, **p < 0.01 compared with healthy controls; ^Δ^*p* < 0.05, ^ΔΔ^*p* < 0.01 compared with ESRD patients without depression.

### 2.3. Sample preparation

Before analysis, every frozen plasma sample was thawed and dissolved at 4 °C. A mixture of acetonitrile/methanol (75:25 *v/v*, 300 μl) (Merck, Germany) was added to plasma (100 μl) to precipitate proteins. After vortexing for 60 s, the mixture was stood for 10 min and then centrifuged at 12,000 *g*/min for 10 min at 4 °C. The supernatant was filtered through 0.22-μm syringe filters (Jinteng, China) and then analyzed by UPLC-MS. Samples were subjected to quality control (QC) by pooling equal volumes of different individual serum samples to assess the reproducibility and reliability of the UPLC-MS system. QC of mixed samples was interspersed at the start, middle, and end of the test.

### 2.4. Liquid chromatography-mass spectrometry (LC-MS) analysis

LC separation was performed on the ZORBAX Eclipse Plus C18 column (2.1 × 100 mm, 3.5 μm; Agilent, USA). The column was maintained at 45 °C. A 10 μl sample was injected into the column for each run in the full loop injection mode. The flow rate of the mobile phase was 0.5 mL/min. Gradient elution was performed with the following solvent system: (A) 0.1% formic acid-water, (B) acetonitrile with 0.1% formic acid. The gradient started with 98% A and decreased to 10% A in 13 min, holding at 10% A for 3 min, and then turned to 98% A immediately, holding at 98% A for 4 min. MS experiments were performed on Triple TOF 5600+, an orthogonal accelerated time of flight mass spectrometer (AB SCIEX, USA) equipped with an electrospray ion source. Data were acquired in positive and negative-V-geometry modes for each LC-MS analysis. The MS parameters were as follows: capillary voltages 2500 and 3000 V, cone gas 50 L/h, desolvation gas 600 L/h, source temperature 120 °C and desolvation temperature 500 °C. The scan range was from 50 to 1500 m/z in the full scan mode and data were collected in centroid mode. Data were centralized during acquisition using independent reference lock-mass ions via the Analyst TF 1.6 and Marker View 1.2.1.

Metabolites were identified by searching the free databases of the Human Metabolome Database (HMDB) [29]. The mass tolerance for the HMDB database search was set at 0.05 Da. The chromatographic retention behavior was used to reduce false-positive matches.

### 2.5. Statistical analysis

The multivariate analyses including unsupervised principal component analysis (PCA), supervised partial least squares discriminant analysis (PLS-DA) and orthogonal projections to latent structures discriminant analysis (OPLS-DA), were conducted to determine the distributions and identify the metabolic difference in two or three groups using the MetaboAnalyst 4.0 [30]. The parameter R2 was used to evaluate the fitting condition of the PLS-DA models, and Q2 was used to assess the predictive ability. These parameters ranged from 0 to 1, where 1 indicated a perfect fit. When the values of R2 and Q2 were >0.5, the model considered to be successful. To avoid overfitting, 7-fold cross-validation and response permutation testing (RPT) were used for model validation [31]. In the PLS-DA model, variables with variable important in projection (VIP) >1 considered to be potentially differential metabolites. Meanwhile, a single variable statistical analysis was performed on the identified metabolites.

Partial Least Squares Discrimination Analysis (PLS-DA) or Orthogonal PLS-DA (OPLS-DA) is a supervised discriminant analysis statistical method. This method uses PLS-DA to establish a model of the relationship between the expression of metabolites and the sample category to realize the prediction of the sample category. Establish a PLS-DA model or OPLS-DA model for group comparisons, and calculate the variable importance for the projection (Variable Importance for the Projection, VIP) to measure the influence of the expression pattern of each metabolite on the classification of each group of samples. And interpretation capabilities, thereby assisting the screening of marker metabolites.

Welch’s *t-*test was used to compare the two groups that were correlated with the intensities of the integrated regions using MetaboAnalyst 4.0, and *p* < 0.05 was considered statistically significant. Moreover, peaks with consistently upregulated or downregulated were identified, the regional intensity data of which were used in hierarchical cluster analysis and metabolic pathway analysis.

### 2.6. Pathway analysis

The differential metabolites were subjected to pathway analysis with Metaboanalyst followed by visualization. Additional powerful pathway enrichment analysis was conducted by Metabolite Set Enrichment Analysis (MSEA). Pearson’s *r* correlation was calculated to evaluate the relations among the biomarkers (*p* < 0.05, impact >0.01).

### 2.7. Receiver operating characteristic (ROC) curve analysis

ROC curve was used to investigate the diagnostic value of differential metabolites. The area under the ROC curve (AUC) indicates the overall ability of the test. A test with an AUC greater than 0.9 has high accuracy, while 0.7–0.9 indicates moderate accuracy, 0.5–0.7 indicates low accuracy and 0.5 a chance result. ROC curve was obtained using the SPSS 25.0 software.

## 3. Results

### 3.1. Biochemical characteristics

The demographics and clinical characteristics of the subjects are shown in Table 1. There was no significant difference in age, gender, and BMI between ESRD patients (with or without depression) and healthy controls. the levels of neutrophilic granulocyte% (N%), systolic blood pressure (SBP), diastolic blood pressure (DBP), triglyceride (TG), creatinine (Cr), blood urea nitrogen (BUN), blood potassium (K^+^), phosphorus and depression scores were significantly increased, while the levels of total cholesterol (TC), hemoglobin (HB) and albumin were significantly decreased in the patients with ESRD (with or without depression) compared with the healthy controls. Moreover, the N% of anxiety score and depression scores were significantly increased in ESRD patients with depression compared with ESRD patients without depression.

### 3.2. Multivariate analysis of UPLC-QTOF/MS

**Figure 1.**
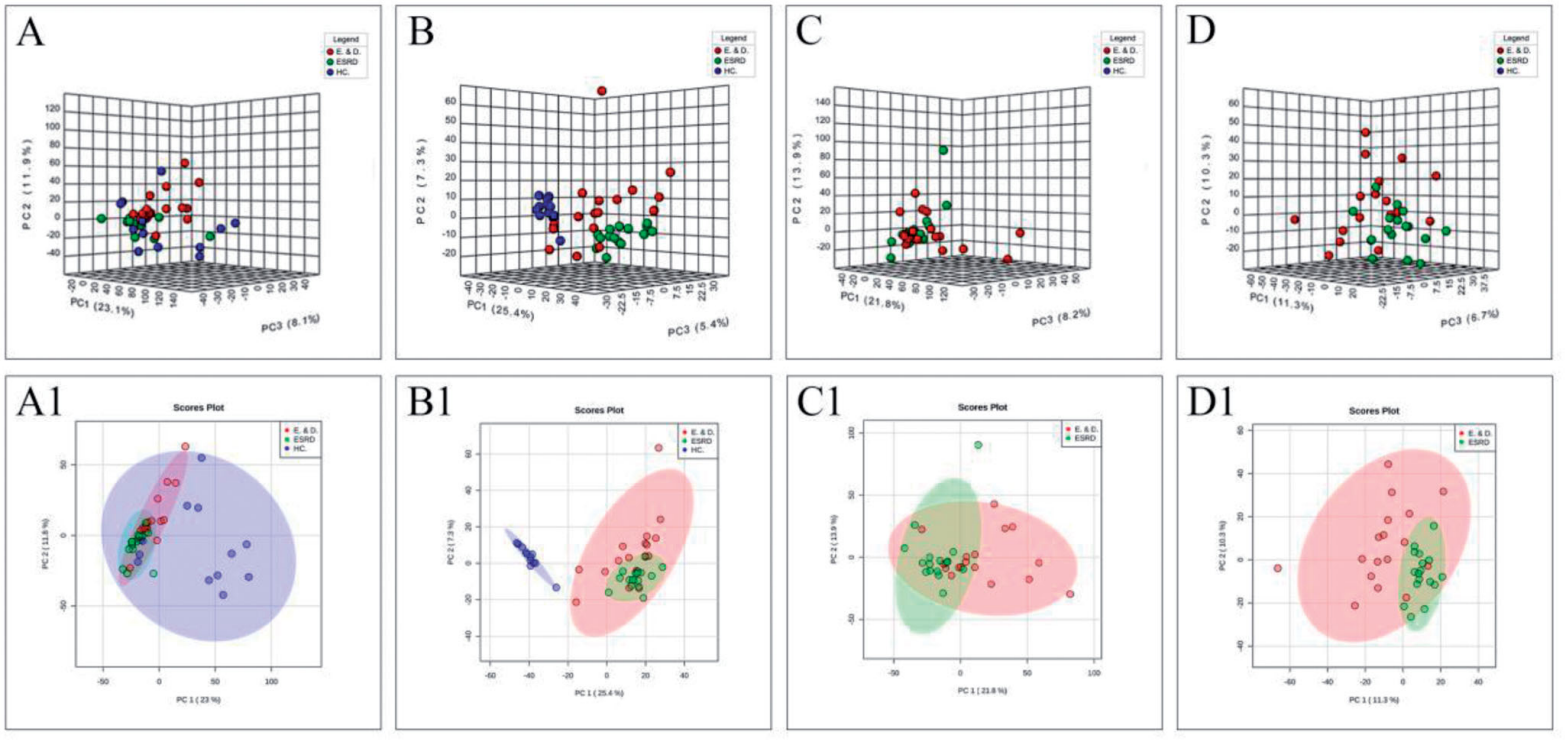
The score plots of PCA model in positive-ion mode (A, C) and negative-ion mode (B, D); A(A1) and B(B1) show health controls, ESRD patients without depression and ESRD patients with depression; C(C1) and D(D1) show ESRD patients without depression and ESRD patients with depression.

The distribution of samples, rationality of the experimental design, and homogeneity of biological replicates were determined by PCA. As shown in the score plot in positive or negative ion modes, ESRD patients without depression were separated from controls, but ESRD patients without depression and with depression were not separated (Figure 1).

**Figure 2.**
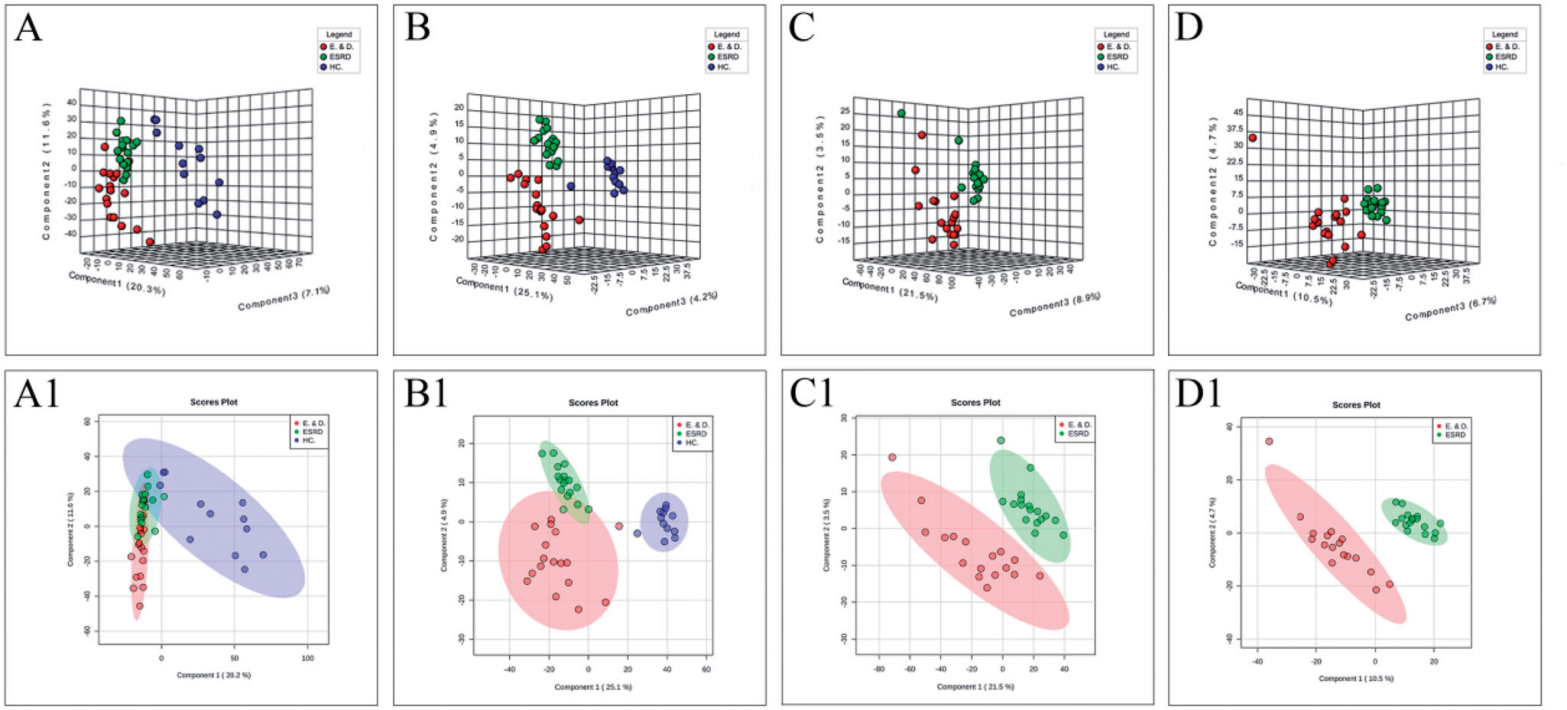
The score plots of PLS-DA model in positive-ion mode (A, C) and negative-ion mode (B, D); A(A1) and B(B1) show health controls, ESRD patients without depression and ESRD patients with depression; C(C1) and D(D1) show ESRD patients without depression and ESRD patients with depression.

**Figure 3.**
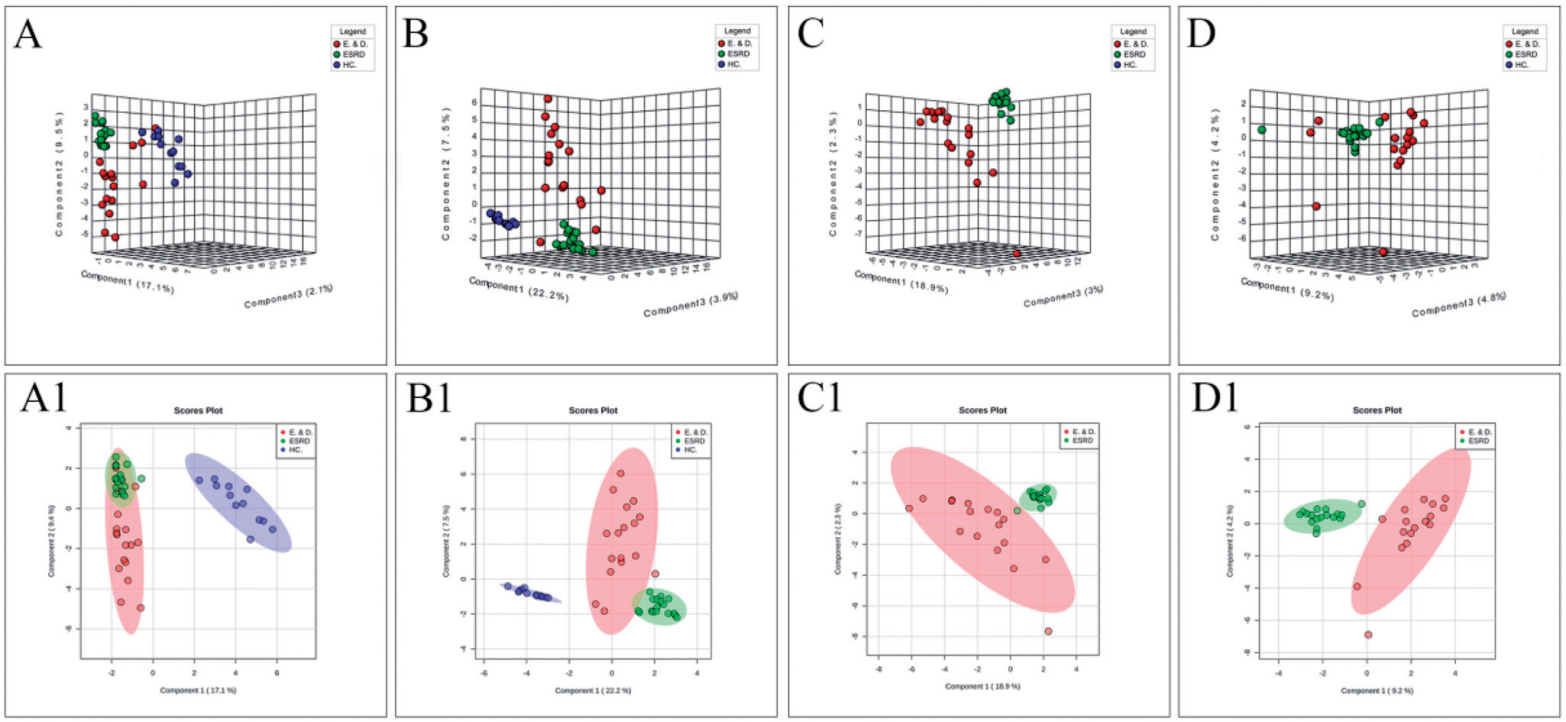
The score plots of the three groups of SPLS-DA model in positive-ion mode (A, C) and negative-ion mode (B, D); A(A1) and B(B1) show health controls, ESRD patients without depression and ESRD patients with depression; C(C1) and D(D1) show ESRD patients without depression and ESRD patients with depression.

To improve the separation of the three groups, PLS-DA and SPLS-DA were performed to visualize their metabolic differences. The score plots of PLS-DA are shown in Figure 2. After the response replacement test of these score plots, there was no overfitting, indicating that the PLS-DA model was successfully constructed. Moreover, in both positive- and negative-ion modes, the three groups were well separated in the SPLS-DA score plot, especially in ESRD patients without depression and ESRD patients with depression (Figure 3). All models were cross-validated and no overfitting was identified. These results indicated that ESRD patients without depression and ESRD patients with depression had different metabolic characteristics.

### 3.3. Differential metabolite analysis and identification

According to the VIP values of characteristic variables obtained from the cross-validated OPLS-DA model, the potential markers were further screened. Variables with FC >5 or FC <0.1, VIP >1 and *p* < 0.05 were considered as potential markers and were structurally identified. A total of 643 ions that were differential between ESRD patients and healthy control. 459 ions in ESRD patients with depression vs. healthy controls, 57 metabolites in ESRD patients without depression vs. ESRD patients with depression were identified. The differential metabolites were mapped to HMDB to identify specific substances. Qualitative results and related information of differential metabolites were screened in positive and negative ion modes. Table 2 showed the results of ESRD patients without depression vs. ESRD patients with depression.

**Table 2.** Characteristic compounds of ESRD patients without depression vs. ESRD patients with depression.

Compound name	ESI^+/-^	FC	Raw.pval	Vip	Trend
5′-Deoxy-5-fluorocytidine	+	5.44E + 00	2.63E-02	2.0509	↑
Dehydrogenated ticlopidine	+	3.12E + 01	1.91E-02	2.1539	↑
N-Desmethylpromazine	+	1.21E-03	1.54E-02	2.2204	↓
Leucyl-phenylalanine	+	3.97E-02	3.61E-02	1.9432	↓
Cladribine	+	7.96E + 00	4.88E-02	1.8341	↑
Pregnenolone	+	2.99E-03	4.59E-02	1.8561	↓
Bevantolol	+	3.23E-02	4.65E-02	1.8518	↓
Tetracosanoic acid	+	9.62E + 03	2.29E-02	2.0964	↑
6-Thioguanosine monophosphate	+	2.33E + 01	4.91E-02	1.8318	↑
Farnesyl pyrophosphate	+	6.39E + 00	3.84E-02	1.9205	↑
Alfuzosin	+	1.89E-02	6.96E-03	2.4468	↓
Cephapirin	+	1.58E + 01	2.22E-02	2.1062	↑
Thiamin pyrophosphate	+	8.73E + 00	2.14E-02	2.1175	↑
Cabergoline	+	1.12E-02	2.37E-02	2.0853	↓
Almitrine	+	9.89E-02	4.63E-02	1.853	↓
Probucol	+	2.65E-02	2.12E-02	2.121	↓
LysoPC(22:4)	+	4.05E-02	4.55E-02	1.86	↓
PA(34:2)	+	2.27E-02	3.31E-03	2.6358	↓
PG(36:1)	+	1.43E-03	4.15E-02	1.8936	↓
CL(i-36:0/18:2)	+	6.71E-02	7.02E-03	2.4442	↓
Phosphoric acid	−	4.63E-02	2.38E-02	1.893	↓
4-Methylcatechol	−	4.15E-02	2.90E-02	1.8329	↓
Dihydrothymine	−	2.92E + 01	1.68E-04	2.9428	↑
3-Methyl-2-oxovaleric acid	−	9.25E-02	6.13E-03	2.2528	↓
Urocanic acid	−	9.61E-02	4.87E-02	1.6664	↓
5-ethyl-5-methyl-2,4-oxazolidinedione	−	4.65E-09	3.66E-02	1.76	↓
L-Glutamic acid	−	2.44E + 01	4.10E-04	2.7978	↑
Gentisic acid	−	2.57E-09	4.69E-02	1.6791	↓
2,5-Furandicarboxylic acid	−	7.19E-09	3.21E-02	1.8021	↓
L-3-Phenyllactic acid	−	7.29E + 00	4.87E-02	1.666	↑
Uric acid	−	1.42E-09	4.02E-02	1.7302	↓
N-Acetyl-L-aspartic acid	−	2.37E-02	4.40E-03	2.3306	↓
Serotonin	−	2.28E-02	1.74E-02	1.9826	↓
Vanylglycol	−	4.72E-03	2.77E-02	1.8473	↓
2-Phosphoglyceric acid	−	5.67E-02	2.84E-03	2.4274	↓
Homocitrulline	−	6.07E + 00	2.78E-03	2.4319	↑
Hydroxyphenylacetylglycine	−	6.80E + 00	7.71E-03	2.1971	↑
1-Hydroxylorcaserin	−	1.32E-09	2.63E-02	1.8631	↓
Propenoylcarnitine	−	2.18E-09	4.28E-02	1.7091	↓
cyclic 6-Hydroxymelatonin	−	3.23E + 01	4.43E-04	2.7843	↑
Desacetyl-nitazoxanide	−	1.06E + 01	1.96E-02	1.9492	↑
Deoxyartemsinin	−	1.77E + 01	3.81E-04	2.8099	↑
Malaoxon	−	5.64E-02	1.52E-02	2.0206	↓
5-HEPE	−	7.48E-02	1.90E-02	1.9573	↓
15(S)-Hydroxyeicosatrienoic acid	−	2.00E-02	2.33E-02	1.8993	↓
5′-O-Desmethyl omeprazole	−	4.06E-02	4.61E-04	2.7776	↓
17-HDoHE	−	8.61E-02	1.29E-02	2.0645	↓
5-(4′-Hydroxyphenyl)-gamma-valerolactone-4′-O-glucuronide	−	8.97E + 00	2.34E-02	1.8971	↑
Hydromorphone-3-sulphate	−	5.47E + 00	4.59E-02	1.686	↑
6-Thioxanthine 5′-monophosphate	−	8.21E-02	7.36E-03	2.2084	↓
Hydroxylansoprazole	−	8.92E-09	4.68E-02	1.6799	↓
Eletriptan N-oxide	−	6.01E-02	2.20E-02	1.9162	↓
Carboxycelecoxib	−	6.03E-02	3.38E-02	1.7851	↓
Zileuton O-glucuronide	−	2.53E-09	1.50E-02	2.0241	↓
Hesperetin 3′-O-glucuronide	−	7.95E + 00	3.72E-02	1.7551	↑
DG(30:1)	−	7.01E + 00	8.44E-03	2.1747	↑
Enkephalin L	−	1.01E + 01	3.51E-02	1.7733	↑

Table 2 shows the qualitative results and related information of screening differential metabolites in the positive- and negative-ion mode.

### 3.4. Clustering analysis

**Figure 4.**
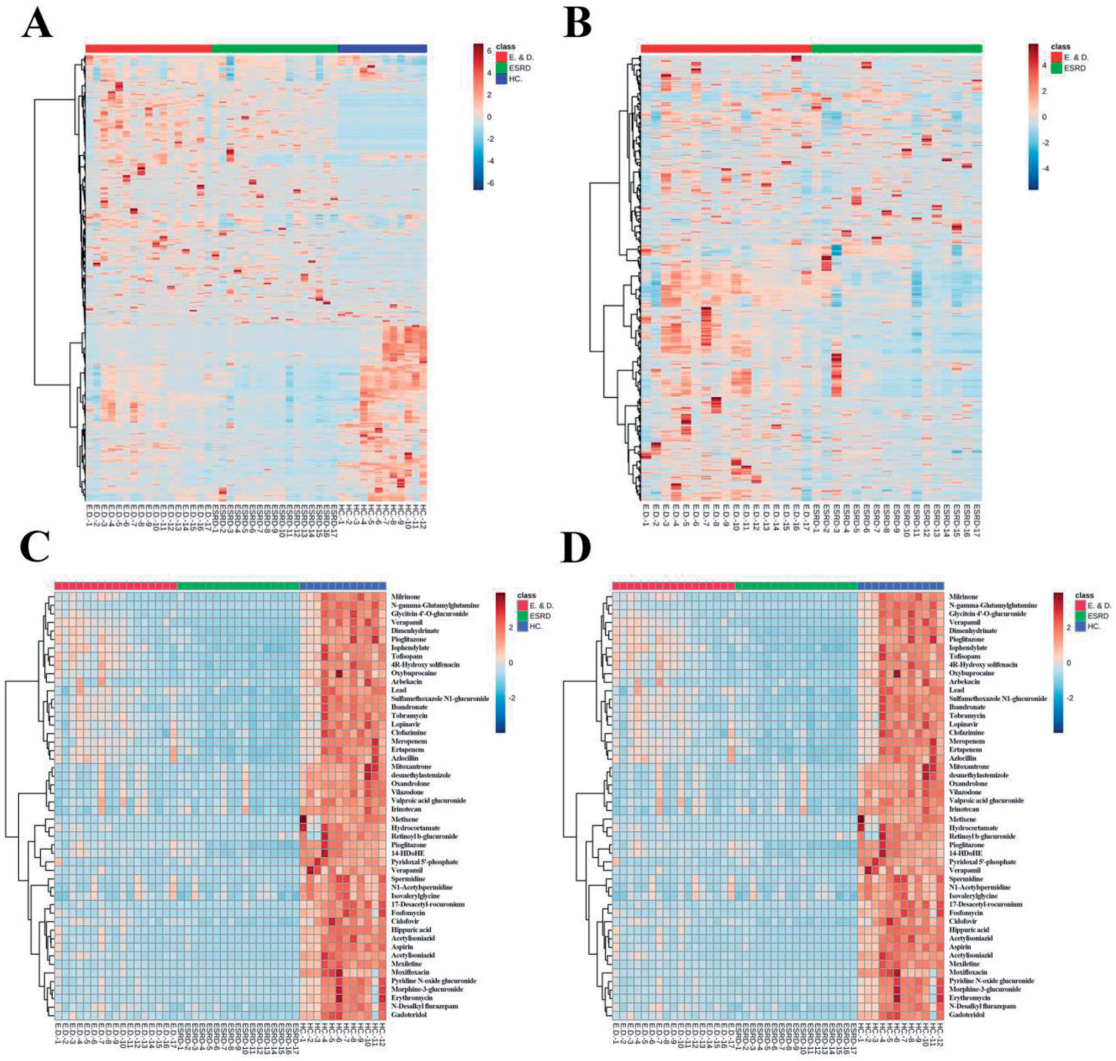
Thermogram of the relative content of differential metabolites: (A) is three groups; (B) shows ESRD patients without depression and ESRD patients with depression. (C) Shows the analytical heat map of the 50 metabolite contents with the largest differences between the three groups. (D) Shows the analytical heat map of the 50 metabolite contents with the largest difference between the two groups.

The heatmaps of the differential metabolites are shown in Figure 4, which provide an intuitive understanding of the relative content of each metabolite. Bidirectional clustering of samples and metabolites was performed on all metabolites using the hierarchical clustering analysis. Clustering analysis for the metabolomics data, using a dendrogram accompanied the head map, has the potential for estimating relationships among these differential metabolites.

### 3.5. Pathway analysis

**Figure 5.**
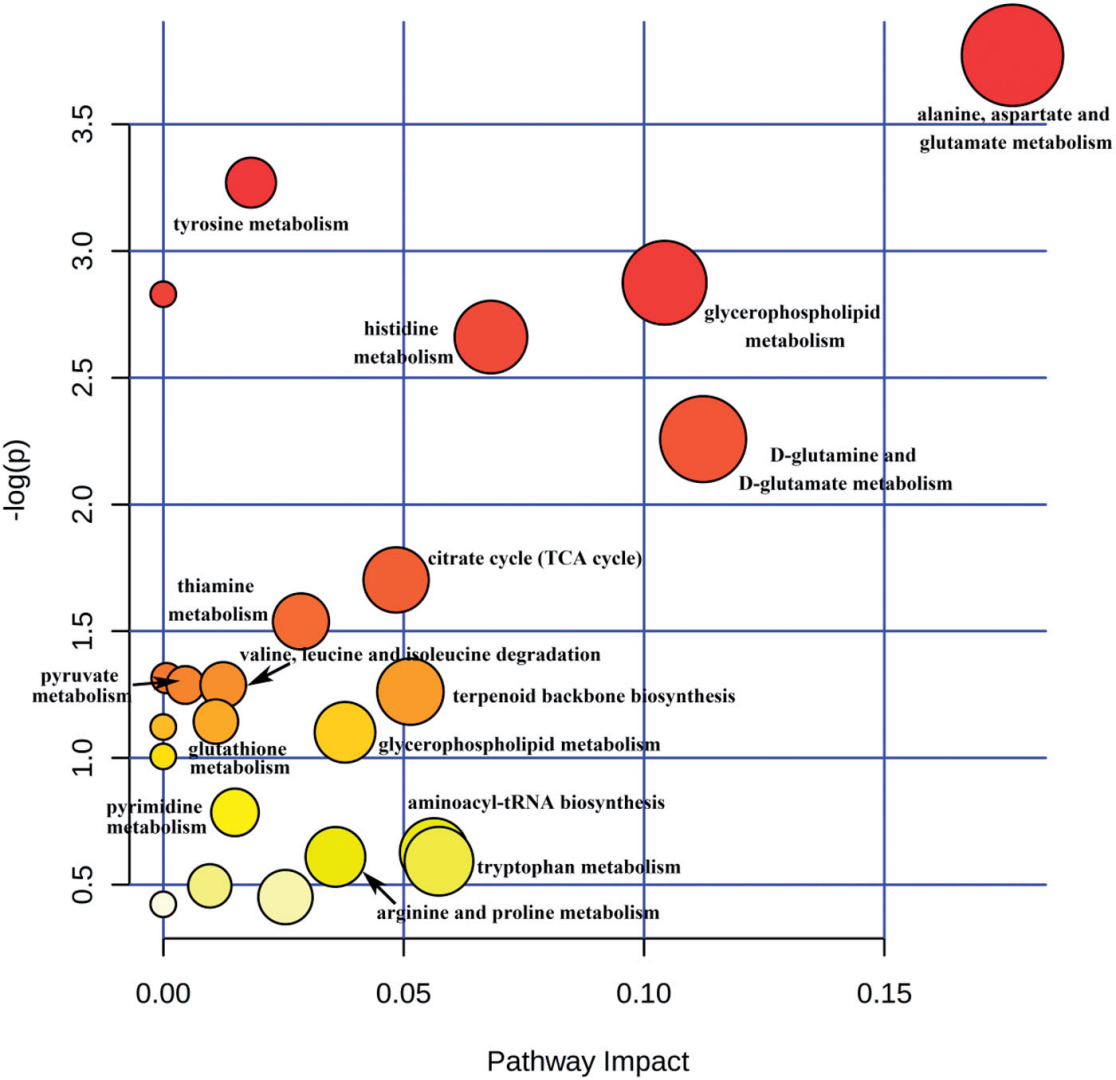
Scores of metabolite pathways involved by differential metabolites in ESRD patients without depression vs. ESRD patients with depression. The size and color of each circle are based on pathway impact value and *p* value, respectively.

A pathway analysis of differential compounds was performed using the KEGG database. The relevant influence scores (-log(p) >0.5, impact >0.01) of metabolic pathways enriched by the differential compound in ESRD patients without depression vs. ESRD patients with depression are shown in Table 3. For simplicity, the metabolic pathways were converted into a pathway overview map, each point representing one pathway (Figure 5). The abscissa is the importance value of the compound in the pathway, and the ordinate is the negative logarithm of the *p*-value log with a base of 10; the closer to the upper right corner, the more significant the enrichment represents and the more important the role of the compound plays in the pathway. There were 19 differential serum metabolite pathways in ESRD patients without depression vs. ESRD patients with depression, and 16 of them were key pathways.

**Table 3.** Different metabolite pathway in ESRD patients without depression versus ESRD patients with depression.

	Total	Expected	Hits	Raw *p*	Holm adjust	FDR	Impact
Alanine, aspartate and glutamate metabolism	24	0.2393	2	0.02301	1	1	0.17664
Tyrosine metabolism	76	0.75779	3	0.038024	1	1	0.01825
Glycerophospholipid metabolism	39	0.38887	2	0.056434	1	1	0.10429
Histidine metabolism	44	0.43872	2	0.069917	1	1	0.06816
D-Glutamineand D-glutamate metabolism	11	0.10968	1	0.10458	1	1	0.11230
Citrate cycle (TCA cycle)	20	0.19942	1	0.18227	1	1	0.04844
Thiamin metabolism	24	0.23930	1	0.21468	1	1	0.02865
Glycolysis or Gluconeogenesis	31	0.30910	1	0.26847	1	1	0.00068
Pyruvate metabolism	32	0.31907	1	0.27586	1	1	0.00459
Glycerolipid metabolism	32	0.31907	1	0.27586	1	1	0.01247
Terpenoid backbone biosynthesis	33	0.32904	1	0.28317	1	1	0.05141
Glutathione metabolism	38	0.37889	1	0.31871	1	1	0.01095
Valine, leucine and isoleucine degradation	40	0.39884	1	0.33245	1	1	0.03781
Pyrimidine metabolism	60	0.59826	1	0.45599	1	1	0.01492
Aminoacyl-tRNA biosynthesis	75	0.74782	1	0.53393	1	1	0.05634
Arginine and proline metabolism	77	0.76776	1	0.54348	1	1	0.03582
Tryptophan metabolism	79	0.78770	1	0.55284	1	1	0.05734
Purine metabolism	92	0.91732	1	0.60933	1	1	0.00969
Steroid hormone biosynthesis	99	0.98712	1	0.63685	1	1	0.02542

Table 3 shows the relative impact scores of metabolic pathways enriched by different compounds in ESRD patients with or without depression.

### 3.6. ROC analysis

To obtain a simple metabolite combination that can separate ESRD patients and ESRD patients with depression in clinical practice, we further analyzed the 57 differential metabolites in the main metabolic pathway. To investigate the diagnostic value of these differential metabolites, ROC curve analysis was conducted to assess the sensitivity and specificity of these metabolites. The compounds with AUC >0.90 are shown in Table 4.

**Table 4.** The metabolites for the diagnosis of ESRD with depression (AUC ≥0.90).

Compound	AUC	TPR	FPR	95% CI
5-ethyl-5-methyl-2,4-oxazolidinedione	0.934	0.9	0.8	0.834–0.986
Gentisic acid	0.965	0.9	0.9	0.896–1.000
2,5-Furandicarboxylic acid	0.931	0.9	0.8	0.829–0.986
Uric acid	0.931	0.9	0.8	0.834–0.990
Serotonin	0.931	0.9	0.9	0.822–0.986
2-Phosphoglyceric acid	0.924	0.8	0.9	0.811–0.986
N-Desmethylpromazine	0.965	0.9	0.9	0.900–1.000
Leucyl-phenylalanine	0.938	0.9	0.9	0.834–0.990
Propenoyl carnitine	0.934	0.9	0.8	0.832–0.990
Malaoxon	0.927	0.9	0.8	0.803–0.990
Pregnenolone	0.976	0.9	0.9	0.917–1.000
Bevantolol	0.948	0.9	0.9	0.861–1.000
6-Thioxanthine 5′-monophosphate	0.924	0.9	0.9	0.789–1.000
Hydroxyl ansoprazole	0.924	0.9	0.8	0.824–0.986
Alfuzosin	0.931	0.8	0.9	0.823–0.986
Zileuton O-glucuronide	0.958	0.9	0.9	0.867–1.000
Cabergoline	0.952	0.9	0.8	0.849–0.997
Probucol	0.931	0.9	0.8	0.815–0.986
PA(34:2)	0.945	0.9	0.9	0.839–1.000
PG(36:1)	0.955	0.9	0.9	0.875–1.000

TPR: true-positive rate; FPR: false-positive rate; 95% CI: 95% confidence interval.

## 4. Discussion

**Figure 6.**
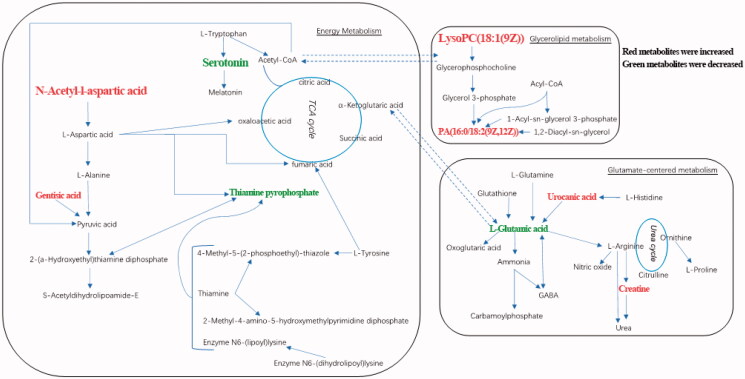
Biochemical transformation of differential metabolites. ESRD patients with depression compared with ESRD patients without depression, red metabolites have increased and green metabolites decreased.

This study examined serum metabolite differences between ESRD patients/ESRD patients with depression and healthy controls, and between ESRD patients and ESRD patients with depression. Based on the pattern recognition method and the recognition model (PLS-DA, OPLS-DA), metabolite changes between groups were distinguished, and satisfactory model parameters were obtained. Through multivariate and univariate statistical analyses, the unique metabolic patterns related to ESRD were also obtained. The differential metabolites in ESRD patients and healthy controls were involved in metabolic pathways such as alanine, aspartate and glutamate metabolism, phenylalanine metabolism, glutathione metabolism, and cysteine and methionine metabolism. ESRD patients with depression were significantly different from ESRD patients without depression in metabolic pathways, such as energy metabolism, glycerolipid metabolism and glutamate-centered metabolism (Figure 6). Additionally, differential metabolites with high diagnostic performance may serve as potential diagnostic markers for distinguishing ESRD patients with or without depression.

### 4.1. Metabolic disorders in ESRD patients

#### 4.1.1. Clinical biochemical characteristics of ESRD patients

Compared with the healthy control group, the levels of SBP, DBP, TG, Cr, BUN, K^+^, phosphate and depression scores of the ESRD patients without depression were significantly increased, while HB and albumin were significantly decreased. These biochemical changes were consistent with hypertension, azotemia, and anemia in ESRD patients, and suggested a high inflammatory response in ESRD patients.

#### 4.1.2. Reduction of antioxidants

Glutathione, taurine and hypotaurine metabolism, as well as cysteine and methionine metabolic pathways, are abnormal in ESRD patients and metabolites in these pathways such as S-adenosyl methionine (SAM), glutathione (GSH) and taurocholic acid antioxidants were lower than that of the healthy controls. The GSH structure contains an active sulfhydryl group (–SH) that is easily oxo-dehydrogenated; studies have shown that SAM inhibits the strong inflammatory and oxidative stress processes that occur in patients [32]. Therefore, ESRD patients have reduced antioxidant capacity and may have oxidative stress damage *in vivo*, which is consistent with the previous findings [33,34].

#### 4.1.3. Metabolic disorders of aromatic amino acids

Phenylalanine, tyrosine, and tryptophan belong to aromatic amino acids, in which phenylalanine is catalyzed by phenylalanine hydroxylase to form tyrosine, and tyrosine is further metabolized to produce catecholamine (dopamine, norepinephrine, and epinephrine) [35]. Compared with the healthy controls, the tyrosine content of the ESRD patients without depression was significantly decreased, which was consistent with previous studies [36], and the significantly decreased tyrosine level was also observed in patients and animal models with CKD [37–39]. In addition, patients with ESRD had a significantly decreased kynurenine level and a significantly increased 3-hydroxyanthranilic acid (3-HANA) level than healthy controls [40]. Tryptophan is mainly metabolized by the kynurenine pathway and the serotonin metabolic pathway, the former being more than 95% in mammals [41]; kynurenine can inhibit antigen presentation, suppress the immune response, and ultimately reduce inflammation [42]. However, 3-HANA is neurotoxic and induces the formation of free radicals such as hydroxyl radicals and hydrogen peroxide, and raises the level of oxidative stress [43]. It is concluded that ESRD may be in a state of a high inflammatory response and oxidative stress [44,45].

### 4.2. Metabolic disorders in ESRD patients with depression

#### 4.2.1. Clinical biochemical characteristics of ESRD patients with depression

In our study, the neutrophil percentage of the ESRD patients with depression was higher than that of the ESRD patients without depression. The occurrence of depression is highly related to inflammation [46–48], which is shown obviously in ESRD patients, with neutrophils as the indicator of inflammatory response [49]. It was been reported that the presence of inflammatory factors such as TNF-α, IL-4, IL-6 in patients ESRD may be effectively regulated by the hypothalamic–pituitary–adrenal axis (HPA); Inflammatory factors can also directly stimulate HPA to cause abnormalities. Studies have shown that HPA abnormalities are one of the main causes of depression [50]. Therefore, high inflammatory response in ESRD patients with depression may be the pathological basis of depression.

#### 4.2.2. Energy metabolism

Compared with ESRD patients without depression, ESRD patients with depression had higher N-acetyl-L-aspartic acid (NAA) and gentisic acid, and lower serotonin and thiamin pyrophosphate (TPP). KEGG analysis showed that these metabolites were involved in the TCA cycle. TCA cycle is the ultimate and hub metabolic pathway of three major nutrients (sugars, lipids, and amino acids) [51]. Dysregulation of TCA metabolism has been reported in ESRD patients with depression [27]. The disorder of nutrient metabolism is common in ESRD patients, leading to insufficient energy supply for biochemical reactions.

NAA as a biomarker of neuronal damage severity and only exists in neurons, which is one of the most concentrated metabolites in the human brain and is not detected in the blood [52]. This study revealed that the NAA level in ESRD patients with depression was higher than that in ESRD patients without depression, which may be due to neuronal apoptosis and necrosis, indicating that neuronal activity was reduced or functional damage in ESRD patients with depression. Serotonin is an important neurotransmitter, and the lack of serotonin in the central nervous system can result in depression. The reduction of serotonin function and activity is closely related to depression, loss of appetite, and endocrine dysfunction [53]. This phenomenon can be observed in patients with major depression [29]. In accordance with the findings above, our result showed that the serotonin level in ESRD patients with depression was lower than that in ESRD patients. We speculated that the factors affecting the metabolism of tryptophan to serotonin and further metabolism to melatonin or acetyl-CoA in ESRD patients with depression may be one of the causes of depression.

#### 4.2.3. Glycerolipid metabolism

In this study, we also found abnormalities in glycerolipid metabolism. Elevated levels of LysoPC(18:1), PG(36:1) and PA(34:2) are observed in ESRD patients with depression compared with ESRD patients without depression. Phospholipids that account for 60% of the brain weight, is critical for brain neuronal structures especially synaptic structures [54]. The three phospholipids of PA, PG, and LysoPC play important roles in signal transduction of dopamine, serotonin, glutamate, and acetylcholine [55,56]. The dysregulation of lipid metabolism has been demonstrated in patients and rats with CKD and ESRD [57–59]. It has been reported that PA, PG, and LysoPC are important signaling molecules with various biological functions involved in cell proliferation and inflammatory processes [60–62]. Therefore, our current demonstrated the dysregulation of lipid metabolism in ESRD patients with depression.

## 5. Limitation

This study only explored depressed or nondepressed ESRD patients through nontargeted metabolomics. The conclusions that can be explained are limited. A number of studies have shown that in addition to inflammation that directly stimulates HPA, cachexia caused by chronic inflammation related to latent infection or malignant disease may also participate in the development of depression in patients with ESRD. Therefore, further experiments and data are needed to explain the relationship between the course of ESRD and depression in patients.

## 6. Conclusions

Our research uses non-targeted metabolomics methods to study the metabolic characteristics of ESRD patients and ESRD patients with depression. Inflammation, oxidative stress and abnormal energy metabolism are related to the pathogenesis of patients with ESRD depression, which may be a promising target for treatment. In addition, several metabolites have been found, and they may play an important role in the development of depression in ESRD patients.

## Data Availability

The study data can be accessed from the corresponding author Ran J.H. or Li J.F. by request.

## Abbreviations

α-KGDHC: alpha-ketoglutarate dehydrogenase complex
ATP: adenosine triphosphate
AUC: the area under the curve
BCKDH: branched-chain alpha-keto acid dehydrogenase complex
BUN: blood urea nitrogen
Cr: creatinine
CKD: chronic kidney disease
Cr: creatinine
DBP: diastolic blood pressure
ESRD: end stage renal disease
FC: fold change
GAD: glutamic acid decarboxylase
HAMD: Hamilton Depression Scale for depression
HB: hemoglobin
HMDB: human metabolome database
HPA: hypothalamic–pituitary–adrenal axis
IL-4: interleukin-4
KEGG: Kyoto Encyclopedia of Genes and Genomes
N%: neutrophilic granulocyte%
NAA: N-acetyl-L-aspartic acid
OPLS-DA: orthogonal partial least square-discriminate analysis
PCA: principal component analysis
PDHC: pyruvate dehydrogenase complex
PLS-DA: partial least squares-discriminant analysis
PUFAs: polyunsaturated fatty acids
RBC: red blood cell
RPT: response permutation testing
ROC: receiver operating curve
SBP: systolic blood pressure
SSRIs: selective serotonin reuptake inhibitors
TCA-cycle: tricarboxylic acid cycle
TC: total cholesterol
TG: triglyceride
TPP: thiamin pyrophosphate
UPLC-QTOF/MS: ultra-high-performance liquid chromatography tandem quadrupole time-of-flight mass spectrometry
VIP: variable important in projection
WBC: white blood cell.
